# Dual tracer tau PET imaging reveals different molecular targets for ^11^C-THK5351 and ^11^C-PBB3 in the Alzheimer brain

**DOI:** 10.1007/s00259-018-4012-5

**Published:** 2018-05-12

**Authors:** Konstantinos Chiotis, Per Stenkrona, Ove Almkvist, Vladimir Stepanov, Daniel Ferreira, Ryosuke Arakawa, Akihiro Takano, Eric Westman, Andrea Varrone, Nobuyuki Okamura, Hitoshi Shimada, Makoto Higuchi, Christer Halldin, Agneta Nordberg

**Affiliations:** 1 Department of Neurobiology, Care Sciences and Society, Center for Alzheimer Research, Translational Alzheimer Neurobiology, Karolinska Institutet, Stockholm, Sweden; 2 Department of Clinical Neuroscience, Center for Psychiatric Research, Karolinska Institutet and Stockholm County Council, Stockholm, Sweden; 3 Theme Aging, Karolinska University Hospital, Stockholm, Sweden; 4 Department of Psychology, Stockholm University, Stockholm, Sweden; 5 Department of Neurobiology, Care Sciences and Society, Center for Alzheimer Research, Division of Clinical Geriatrics, Karolinska Institutet, Stockholm, Sweden; 6 Cyclotron and Radioisotope Center, Tohoku University, Sendai, Japan; 7 Division of Pharmacology, Faculty of Medicine, Tohoku Medical and Pharmaceutical University, Sendai, Japan; 8 National Institute of Radiological Sciences, National Institutes for Quantum and Radiological Science and Technology, Chiba, Japan

**Keywords:** Tau, Neurofibrillary tangles, Amyloid-beta, Alzheimer’s disease, PET imaging, Neurodegeneration

## Abstract

**Purpose:**

Several tau PET tracers have been developed, but it remains unclear whether they bind to the same molecular target on the heterogeneous tau pathology. In this study we evaluated the binding of two chemically different tau-specific PET tracers (^11^C-THK5351 and ^11^C-PBB3) in a head-to-head, in vivo, multimodal design.

**Methods:**

Nine patients with a diagnosis of mild cognitive impairment or probable Alzheimer’s disease and cerebrospinal fluid biomarker evidence supportive of the presence of Alzheimer’s disease brain pathology were recruited after thorough clinical assessment. All patients underwent imaging with the tau-specific PET tracers ^11^C-THK5351 and ^11^C-PBB3 on the same day, as well as imaging with the amyloid-beta-specific tracer ^11^C-AZD2184, a T1-MRI sequence, and neuropsychological assessment.

**Results:**

The load and regional distribution of binding differed between ^11^C-THK5351 and ^11^C-PBB3 with no statistically significant regional correlations observed between the tracers. The binding pattern of ^11^C-PBB3, but not that of ^11^C-THK5351, in the temporal lobe resembled that of ^11^C-AZD2184, with strong correlations detected between ^11^C-PBB3 and ^11^C-AZD2184 in the temporal and occipital lobes. Global cognition correlated more closely with ^11^C-THK5351 than with ^11^C-PBB3 binding. Similarly, cerebrospinal fluid tau measures and entorhinal cortex thickness were more closely correlated with ^11^C-THK5351 than with ^11^C-PBB3 binding.

**Conclusion:**

This research suggests different molecular targets for these tracers; while ^11^C-PBB3 appeared to preferentially bind to tau deposits with a close spatial relationship to amyloid-beta, the binding pattern of ^11^C-THK5351 fitted the expected distribution of tau pathology in Alzheimer’s disease better and was more closely related to downstream disease markers.

**Electronic supplementary material:**

The online version of this article (10.1007/s00259-018-4012-5) contains supplementary material, which is available to authorized users.

## Introduction

The aggregation of abnormally hyperphosphorylated tau protein in neurofibrillary tangles and the aggregation of amyloid-beta fibrils in extracellular plaques are the main neuropathological hallmarks of Alzheimer’s disease (AD) [1]. In recent years, families of PET tracers that selectively target tau pathology have been developed, and many research groups have evaluated them in vivo. All tracers have favourable pharmacokinetics, show low binding in young healthy volunteers considered to be devoid of tau pathology and high binding in patients with AD, with a regional pattern strongly resembling the distribution of tau pathology as described by classical autopsy studies in the field [2–4].

**Fig. 1:**
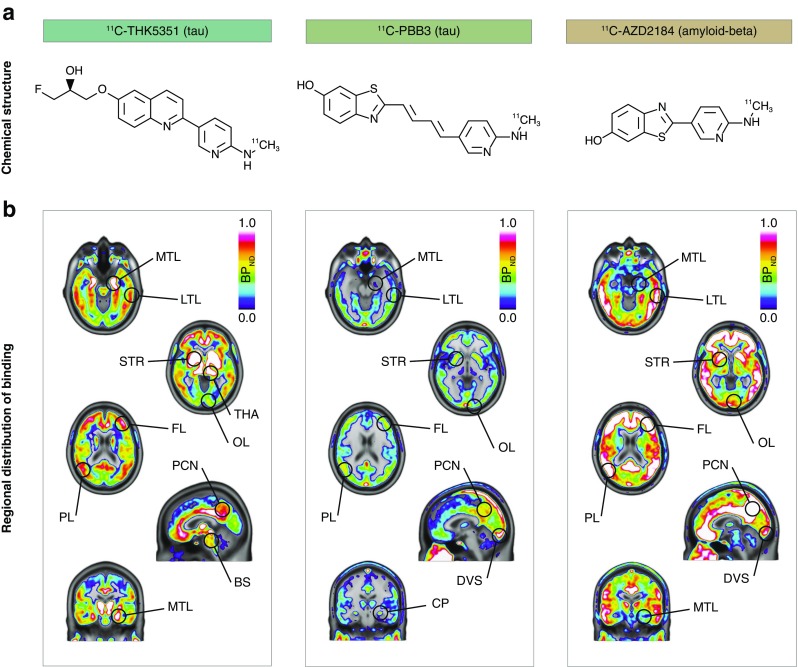
Chemical structures (**a**) and average binding potential (BP_ND_) images (**b**) of ^11^C-ΤΗΚ5351 (tau), ^11^C-PBB3 (tau) and ^11^C-AZD2184 (amyloid-beta) in patients with Alzheimer’s disease (prodromal or dementia; *n* = 9). The results presented were derived from data without correction for the partial volume effect. *BS* brainstem, *CP* choroid plexus, *DVS* dural venous sinus, *FL* frontal lobe, *LTL* lateral temporal lobe, *MTL* medial temporal lobe, *OL* occipital lobe, *PCN* precuneus, *PL* parietal lobe, *STR* striatum, *THA* thalamus

Despite the similar findings reported for all tau PET tracers in different cohorts, the distinct chemical structures of the tracers (Fig. 1a) call into question the similarity of their targets. Indeed, recent in vitro evidence highlights differences in the binding sites of tracers derived from different chemical families, when investigated in the same brain tissue [5, 6]. This should perhaps come as no surprise, since the existing body of research suggests that tau pathology offers a complex target for molecular imaging because of its heterogeneity in terms of tau isoforms affected, conformations adopted, maturation stages of the aggregates and cell types affected [7]. Therefore, although all the developed tracers are designed to target tau, it is likely that their specific targets on tau pathology are substantially different.

The relationships between tau tracer binding and other markers of AD have been assessed, to date, mainly in nonconsecutive studies. The results have indicated that the regional patterns of tau tracer binding are different from those of amyloid-beta tracers, although regional correlations exist [8–11]. Furthermore, the binding of all tau tracers is related to cognitive decline as well as regional neurodegeneration [8, 10–13]; this association becomes more obvious as neurodegeneration advances, as shown by the only longitudinal study [14]. However, since all these investigations were performed in individual cohorts using different tau tracers, it remains unclear whether the relationship between the tracers and the other markers of AD would be different when examined in a head-to-head design.

The aim of this multimodal study was to assess the binding properties of two chemically different tau-specific PET tracers (^11^C-THK5351 and ^11^C-PBB3) in vivo when injected into the same patients on the same day, and to examine their relationship with markers of amyloid-beta deposition, cognitive impairment and measures of cerebrospinal fluid (CSF) tau and medial temporal atrophy.

## Materials and methods

### Study sample

Eleven patients were originally recruited to participate in a cross-sectional, multimodal, head-to-head, in vivo comparison study of the binding characteristics of the tau-specific PET tracers ^11^C-THK5351 and ^11^C-PBB3. All were recruited from the Memory Clinic of the Department of Geriatric Medicine, Karolinska University Hospital, Stockholm, Sweden, where they underwent thorough clinical investigation including medical history, physical examination, laboratory blood tests, apolipoprotein E genotyping, neuropsychological assessment, CSF sampling and structural imaging. The inclusion criteria for the study included objective evidence of cognitive impairment in the neuropsychological assessment and CSF biomarker findings supportive of the presence of AD pathological changes [15]. Seven of the eleven patients fulfilled clinically the Petersen criteria [16] for mild cognitive impairment, while four patients fulfilled the NINCDS-ADRDA [17] and DSM-IV criteria for dementia of the Alzheimer’s type. For the purposes of this study and based on the AD-consistent CSF profile, all patients with mild cognitive impairment were reclassified as having prodromal AD and all patients with AD as having AD dementia [15]. No non-AD-related pathology was detected on MRI in any of the patients, as evaluated by an experienced neuroradiologist at the Karolinska University Hospital. Two patients were excluded because of technical issues during the PET acquisitions.

### Neuropsychological assessment

All participants completed a large battery of neuropsychological tests. The selection of tests was based on previous observations with tau PET imaging [13]. Premorbid cognitive function was assessed with the Swedish National Adult Reading Test (irregularly spelled words; ISW) [18], while current global cognitive function was assessed with the Full Scale Intelligence Quotient (FSIQ), which is based on five subtests from the Weschler Adult Intelligence Scale (Similarities, Information, Block design, Digit span, and Digit symbol) [19]. A summary *z*-score, based on a reference group of healthy controls, was calculated to describe each individual’s cognitive decline from the estimated premorbid cognitive function (decline in FSIQ: premorbid cognitive function, ISW, minus current FSIQ) [20, 21]. Episodic memory performance was assessed using the Rey-Osterrieth Complex Figure (ROCF) delayed recall test, after *z*-score transformation using a reference group of healthy controls [21]. One patient did not complete the ROCF delayed recall test. The Mini-Mental State Examination (MMSE) score was used as a clinical measure of global cognition.

### Image acquisition

^11^C-THK5351 and ^11^C-PBB3 PET measurements were planned for all participants on the same day (median 0 [quartile 1:quartile 3 0:2] days) for imaging tau pathology. An ^11^C-AZD2184 dynamic PET measurement was planned within 2 weeks (3 [3:9] days) for imaging amyloid-beta deposition. A three-dimensional, T1-weighted MRI sequence was performed after a median of 9 [6:67] days to assess medial temporal atrophy.

The ^11^C-THK5351, ^11^C-PBB3 and ^11^C-AZD2184 PET measurements were acquired on a high-resolution research tomograph (HRRT; CTI/Siemens, Knoxville, TN, USA) in list mode, at the Centre for Psychiatric Research, Karolinska Institutet, Stockholm, Sweden. All tracers were synthesized according to standard good manufacturing practice, as described previously [22–24]. The tracer THK5351, although originally developed as an ^18^F tracer, was labelled with ^11^C for the purposes of this project, to allow multitracer imaging on the same day as the ^11^C-PBB3 acquisitions. An individual plaster helmet was made for each participant prior to PET, and was used to minimize head movement during the PET acquisition. For ^11^C-THK5351, 38 frames were acquired over 93 min, and for ^11^C-PBB3 and ^11^C-AZD2184, 33 frames were acquired over 63 min, starting simultaneously with intravenous injection of 350 [322:414] MBq, 343 [300:420] MBq and 308 [295:347] MBq, respectively. The injected mass for each tracer was similar: 0.40 [0.35:0.52] μg, 0.33 [0.20:0.60] μg and 0.20 [0.17:0.44] μg, respectively. A longer acquisition time was used for THK5351 since this was the first in vivo evaluation of the tracer when labelled with ^11^C. The radiosynthesis and injection of ^11^C-PBB3 were carried out without fluorescent lighting to avoid photoisomerization of the tracer [23]. All acquisitions were reconstructed using ordered-subsets expectation maximization.

Three-dimensional, sagittal magnetization-prepared, rapid gradient-echo, T1-weighted MRI sequences were acquired on a 1.5-T Siemens MAGNETOM Avanto imaging system (Siemens AG, Muenchen, Germany) at Praktikertjänst Röntgen Odenplan, Stockholm, Sweden. The parameters applied were as follows: repetition time/echo time/inversion time 1,790/3.53/1,100 ms, field of view 256 × 256 mm, acquisition matrix 256 × 208 mm, flip angle 15°, and slice thickness 1 mm. Full brain and skull coverage was required for the MRI datasets and detailed quality control was carried out on all images according to previously published criteria [25].

### Regions of interest for PET quantification

All individual T1-weighted MRI images were first segmented into six tissue classes (grey matter, white matter, CSF, bone, soft tissue, and air/background) using SPM12 [26]. The inverse nonlinear transformation matrix was used to spatially wrap the anatomical automatic labelling atlas [27] to the individual’s native MRI space. Application of the individual grey matter masks resulted in individual grey matter regions of interest (ROIs). The choice of ROIs for quantifying tracer binding was based on the findings of previously published tau PET imaging studies [8, 11]; the hippocampus, parahippocampal gyrus, fusiform gyrus, inferior temporal gyrus, and medial and lateral occipital lobes (Online Resource 1), and the composite ROIs temporal, frontal, parietal and occipital cortices were selected.

### PET image preprocessing

The individual grey matter ROIs were applied to the dynamic ^11^C-THK5351, ^11^C-PBB3 and ^11^C-AZD2184 images in the native PET space, through an intermediate MRI to PET coregistration step using SPM12, to preserve the high resolution of the PET data. Voxel-based kinetic modelling for all tracers was applied with the wavelet-aided parametric imaging method [28] to obtain high resolution, noise-robust nondisplaceable binding potential (BP_ND_) images over the following measurement intervals: 8–93 min for ^11^C-THK5351, 8–63 min for ^11^C-PBB3 and 45–63 min for ^11^C-AZD2184. The cerebellar cortex was used as the reference region for quantifying the binding of all tracers, a region that has been previously validated against using arterial input function data for both THK5351 and PBB3 [29, 30]. A BP_ND_ isocortical threshold of 0.40 for amyloid-beta positivity was applied to the ^11^C-AZD2184 PET data [31, 32].

THK5351 has no known brain-penetrating metabolites [33] that would affect quantification of the tracer’s binding, but this is not the case for PBB3, which shows such a radiometabolite [23, 34]. Interestingly, however, it has been shown that PBB3 binding can be accurately quantified with simplified reference models, despite the presence of the metabolite, as illustrated using arterial sampling to obtain the parent and metabolite input functions [30]. It is worth noting, however, that in no case can we rule out the possibility that the signal from the radiolabelled PBB3 metabolite is contributing to a certain low degree to the total signal quantified by kinetic models not employing arterial data. In our study, in order validate the voxel-based quantification of ^11^C-THK5351 and ^11^C-PBB3, we carried out region-based kinetic modelling using the reference Logan graphical method [35] and the original multilinear reference tissue model [36] (Online Resource 2), respectively, as proposed previously for each tracer [29, 30, 37].

In order to validate the quantification of tracer binding used in the present work, the dynamic PET data were also corrected for the partial volume effect (PVE) using the geometric transfer matrix method (data are shown in Online Resource 3) [38]. The results presented here in the main body of the text including all main figures were derived from PVE-uncorrected data.

### Cortical thickness measurements

FreeSurfer image processing software, version 6.0 (http://surfer.nmr.mgh.harvard.edu) was used to measure the cortical thickness on T1-weighted MRI images. Cortical reconstruction was performed as described in detail elsewhere [39]. Quality control of the output was carried out. The thickness of the entorhinal cortex was selected in this study as a measure of medial temporal atrophy, based on previous findings with another tau PET tracer [40] showing that entorhinal thickness rather than hippocampal volumes is more closely related to local tau PET tracer binding.

### Cerebrospinal fluid measurements

CSF samples were obtained via lumbar puncture from all patients, under nonfasting conditions, as part of routine memory assessment. Levels of amyloid-beta (Aβ_1–42_), total tau (t-tau), and phosphorylated tau_181p_ (p-tau_181p_) were determined using commercially available sandwich ELISAs (Innogenetics, Ghent, Belgium) at the Clinical Neurochemistry Laboratory, Gothenburg University, Mölndal, Sweden. The Aβ_1–42_, t-tau and p-tau_181p_ reference values used to determine AD-consistent abnormalities in the clinical assessment of the patients were <550 pg/mL, >400 pg/mL and >80 pg/mL, respectively.

### Statistical analysis

Data are presented as number or medians [quartile 1:quartile 3]. Correlations between modalities were analysed using the nonparametric Spearman coefficient. The associations between the tracers in terms of local binding were investigated in the four main lobes and the four temporal ROIs. Associations between tracer binding and age, CSF tau measures, decline in FSIQ and ROCF delayed recall were examined in the temporal ROIs. Associations between entorhinal cortex thickness and local tracer binding were also investigated; the tracer binding was evaluated in the parahippocampal gyrus, since in the atlas used to sample the PET data the entorhinal cortex was included in the parahippocampal gyrus ROI [27]. The association between tracer binding and MMSE results was examined in relation to binding in the four main lobes because of the gross nature of MMSE. The cut-off for statistical significance was *p* < 0.05 (two-tailed). All analyses were carried out using R software, version 3.4.0 (https://www.R-project.org/).

## Results

The characteristics of the study sample are summarized in Table 1. The patients were relatively young (65 [61:70] years), mildly cognitively impaired (MMSE score 27 [25:28]), apolipoprotein E ε4 carriers and amyloid-beta positive based on their ^11^C-AZD2184 PET measurement (isocortical binding 0.91 [0.88:0.95] BP_ND_, cut-off for amyloid-beta positivity 0.40 BP_ND_; for more information see the section PET image preprocessing).

**Table 1:** Demographic and clinical characteristics of the study sample

Characteristic	Value
Clinical characteristics	
Number of participants	9
Gender (M/F)	4/5
Age (years)	65 [61:70]
Education (years)	12 [12:13]
Clinical diagnosis	
Prodromal AD	5
AD dementia	4
APOE carriers	
ε3/ε4	5
ε4/ε4	4
Cognitive performance	
MMSE score	27 [25:28]
Decline in FSIQ based on ISW (*z*-scores)^a^	−2.04 [−0.97:−2.16]
ROCF delayed recall (*z*-scores)^a^	−1.45 [−1.15:−2.09]^c^
CSF biomarkers^b^	
Aβ_1–42_ (pg/mL)	477 [380:539]
Total tau (pg/mL)	548 [450:897]
Phosphorylated tau_181_ (pg/mL)	66 [57:94]

Data are presented as medians [quartile 1:quartile 3] or as number

*Aβ* amyloid-beta, *AD* Alzheimer’s disease, *APOE* apolipoprotein E, *CSF* cerebrospinal fluid, *FSIQ* Full-Scale Intelligence Quotient, *ISW* Irregularly Spelled Words test, *ROCF* Rey-Osterrieth complex figure test

^a^Decline in FSIQ based on ISW and performance in the ROCF delayed recall test are expressed as *z*-scores in comparison with a reference group of healthy controls [21]

^b^The local reference values for Aβ_1–42_, total tau, and phosphorylated tau_181p_ used to determine abnormalities in the clinical assessment of the patients were <550 pg/mL, >400 pg/mL, and >80 pg/mL, respectively

^c^One patient did not complete the ROCF delayed recall test

### Load and regional distribution of tracer binding

**Fig. 2:**
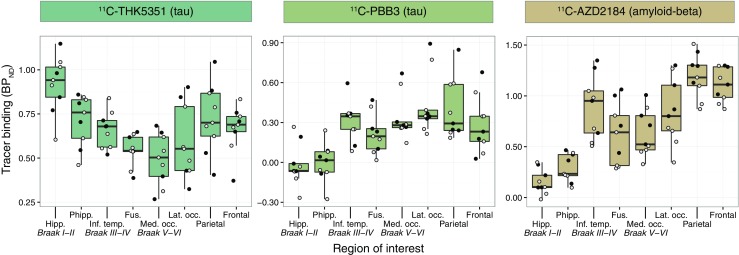
Boxplots illustrating the regional quantification of the binding of ^11^C-ΤΗΚ5351 (tau), ^11^C-PBB3 (tau) and ^11^C-AZD2184 (amyloid-beta) in patients with Alzheimer’s disease (prodromal or dementia; *n* = 9). The results presented were derived from data without correction for the partial volume effect. *Open circles* patients with prodromal Alzheimer’s disease, *closed circles* patients with Alzheimer’s disease dementia; *horizontal lines* median values, *lower and upper hinges* first and third quartiles, *whiskers* range of values excluding potential outliers. *Braak I-VI* regions of interest roughly matching the neuropathological Braak staging system for neurofibrillary tangle pathology for demonstration purposes only [53], *Fus.* fusiform gyrus, *Hipp.* hippocampus, *Inf. temp.* inferior temporal gyrus, *Lat. occ.* lateral occipital cortex, *Med. occ.* medial occipital cortex, *Phipp.* parahippocampal gyrus

Binding of the tau-specific tracers ^11^C-THK5351 and ^11^C-PBB3 was observed in the temporal lobes and other isocortical areas (Fig. 1b). ^11^C-THK5351 showed substantially higher grey matter binding than ^11^C-PBB3. Both tracers showed very low white matter binding. Regional differences in the binding patterns of the two tracers were observed, especially in the temporal lobes. ^11^C-THK5351 binding was higher in the medial than in the lateral temporal lobe, while the opposite pattern was observed for ^11^C-PBB3. Briefly, the highest cortical ^11^C-THK5351 binding was detected in the hippocampus (allocortex), while lower binding was detected in the inferior temporal gyrus, and the lowest cortical binding of the tracer was observed in the medial areas of the occipital lobe (Fig. 2). In contrast, ^11^C-PBB3 showed minimal binding in the hippocampus, and the highest binding in the temporal lobe was seen in the inferior temporal gyrus. The lack of binding of ^11^C-PBB3 in the hippocampus, in contrast to the extensive binding in the adjacent choroid plexus, could be better appreciated on high-resolution PET imaging data (Online Resource 4). ^11^C-THK5351 showed off-target binding in the thalamus and brain stem and ^11^C-PBB3 showed off-target binding in the dural venous sinuses and choroid plexus. Both tracers showed high binding in the striatum, cingulate gyri and precuneus, although this binding was substantially greater for ^11^C-THK5351. PVE correction of tracer binding resulted in higher BP_ND_ values across ROIs for both tracers, although the regional distribution pattern was essentially the same (Online Resource 3).

Online Resource 5 shows the time–activity curves for ^11^C-THK5351 and ^11^C-PBB3 in the participating patients. Both were rapidly taken up by the brain, although uptake of ^11^C-THK5351 was greater and the overall kinetics were faster over the measurement interval compared with ^11^C-PBB3.

In all patients, isocortical binding of the amyloid-beta-specific tracer ^11^C-AZD2184 was widespread, with a binding distribution pattern that was largely distinct from those of ^11^C-THK5351 and ^11^C-PBB3. Interestingly, however, the binding pattern of ^11^C-AZD2184 in the temporal lobe resembled that of ^11^C-PBB3; binding was higher in the lateral temporal lobe than in the medial areas (Figs. 1b and 2). Off-target binding of ^11^C-AZD2184 was observed in the dural venous sinuses.

### Association between tracers with respect to binding

**Fig. 3:**
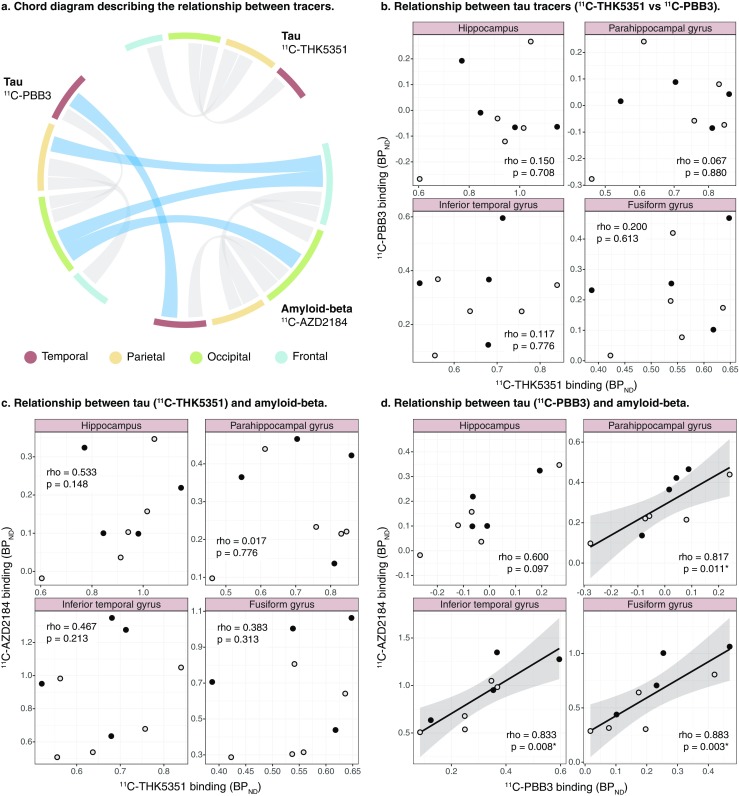
Chord diagram (**a**) and scatterplots (**b–d**) showing the relationships between the binding (BP_ND_) of the tau tracers ^11^C-ΤΗΚ5351 and ^11^C-PBB3, and the amyloid-beta tracer ^11^C-AZD2184. The results presented were derived from data without correction for the partial volume effect. The weight of the strings in the chord diagram represents the strength of the statistically significant Spearman’s regional correlations within or between tracers (*grey and blue strings*, respectively). *Open circles* patients with prodromal Alzheimer’s disease, *closed circles* patients with Alzheimer’s disease dementia (*rho* Spearman’s rho). *p < 0.05

Intriguingly, there were no correlations between ^11^C-THK5351 and ^11^C-PBB3 with respect to binding in the four main lobes or the temporal ROIs (Fig. 3a, b).

There were no correlations between ^11^C-THK5351 and ^11^C-AZD2184 binding across all ROIs (Fig. 3a, c). In contrast, the local binding of ^11^C-PBB3 and of ^11^C-AZD2184 correlated positively in the temporal and occipital lobes (rho = 0.750, *p* = 0.020, and rho = 0.817, *p* = 0.007, respectively; Fig. 3a). In more detail, ^11^C-PBB3 and ^11^C-AZD2184 binding correlated strongly in all temporal sub-ROIs examined, except the hippocampus in which a trend was observed (Fig. 3d).

### Association between tau tracer binding and cognitive performance

**Fig. 4:**
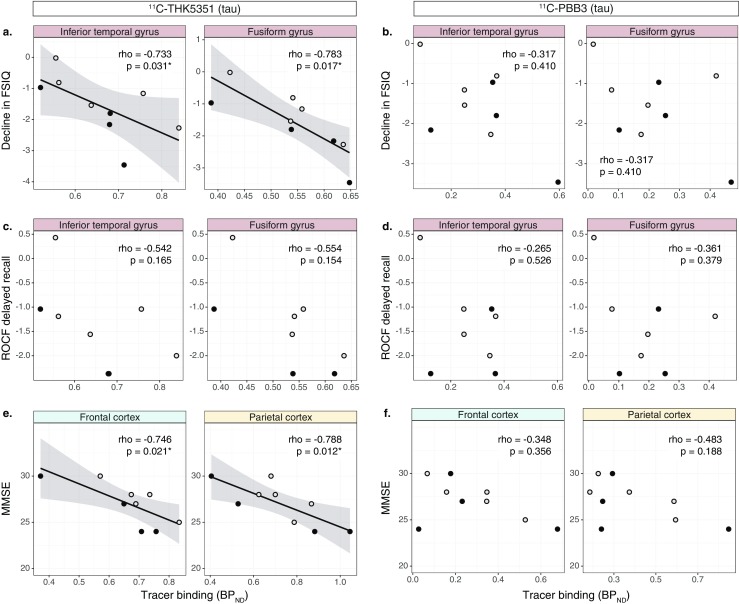
Scatterplots showing the relationships between the binding (BP_ND_) of the tau tracers (^11^C-ΤΗΚ5351 and ^11^C-PBB3) and the decline in FSIQ (global cognition; **a**, **b**), ROCF delayed recall (episodic memory; **c**, **d**) and MMSE (global cognition; **e**, **f**). The declines in FSIQ and ROCF delayed recall are expressed as *z*-scores from comparison with a reference group of healthy controls [21]. The results presented were derived from data without correction for the partial volume effect. *Open circles* patients with prodromal Alzheimer’s disease, *closed circles* patients with Alzheimer’s disease dementia (*rho* Spearman’s rho). *p < 0.05

^11^C-THK5351 binding in the inferior temporal and fusiform gyri correlated negatively with decline in FSIQ (Fig. 4a). ^11^C-THK5351 binding showed a moderate correlation with ROCF delayed recall, although the association did not reach statistical significance (rho = −0.542 to −0.602, *p* = 0.114–0.165; Fig. 4c). No statistically significant correlation was detected between ^11^C-PBB3 binding and decline in FSIQ (Fig. 4b) or ROCF delayed recall test scores (rho = −0.133 to −0.361, *p* = 0.379–0.754; Fig. 4d). ^11^C-THK5351 binding in the frontal and parietal lobes correlated negatively with MMSE score (Fig. 4e). ^11^C-PBB3 binding was also negatively correlated with MMSE score, although the outlier profile of a single patient resulted in a statistically nonsignificant correlation between the two (Fig. 4f). The outlier patient (AD dementia, MMSE score 24) had the poorest education (8 years) of the study sample and clear discordance in the binding of the two tau-specific tracers. The exclusion of this patient resulted in statistically significant negative correlations between ^11^C-PBB3 binding and MMSE score in the frontal, parietal and occipital lobes (*n* = 8, rho = −0.740 to −0.837, *p* = 0.010–0.036).

### Association between tau tracer binding and other markers of disease

**Fig. 5:**
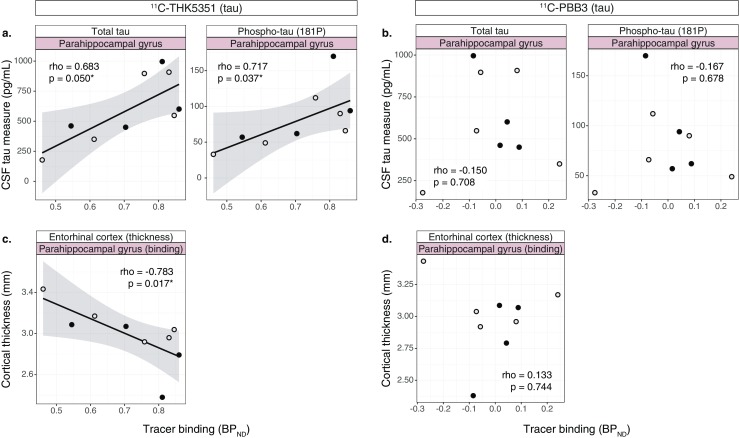
Scatterplots showing the relationships between the binding (BP_ND_) of the tau tracers (^11^C-ΤΗΚ5351 and ^11^C-PBB3) and CSF tau measures (**a**, **b**), and entorhinal cortex thickness (**c**, **d**). The results presented were derived from data without correction for the partial volume effect. *Open circles* patients with prodromal Alzheimer’s disease, *closed circles* patients with Alzheimer’s disease dementia (*rho* Spearman’s rho). *p < 0.05

^11^C-THK5351 binding in the parahippocampal gyrus was significantly positively correlated with CSF tau levels (t-tau and p-tau_181p_; Fig. 5a); the correlation was not significant in the other temporal ROIs examined except for a trend for a significant correlation between ^11^C-THK5351 binding and p-tau_181p_ in the inferior temporal gyrus (rho = 0.617, *p* = 0.086). In contrast, there were no significant correlations between ^11^C-PBB3 binding and CSF tau levels (Fig. 5b).

^11^C-THK5351 binding in the parahippocampal gyrus correlated negatively with entorhinal cortex thickness (rho = −0.783, *p* = 0.017), but there was no correlation between ^11^C-PBB3 binding and cortical thickness in the same ROI (Fig. 5c, d).

### Association between tau tracer binding and age

**Fig. 6:**
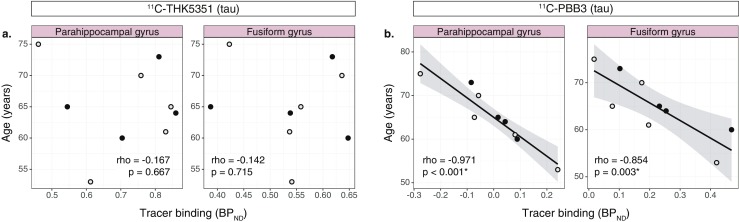
Scatterplots showing the relationships between the binding (BP_ND_) of the tau tracers (^11^C-ΤΗΚ5351 and ^11^C-PBB3) and age (**a**, **b**). The results presented were derived from data without correction for the partial volume effect. *Open circles* patients with prodromal Alzheimer’s disease, *closed circles* patients with Alzheimer’s disease dementia (*rho* Spearman’s rho). *p < 0.05

There were strong negative correlations between ^11^C-PBB3 binding and age in all temporal ROIs examined (rho = −0.812 to −0.971, *p* < 0.01; Fig. 6b). No correlations were detected between ^11^C-THK5351 binding and age in the temporal ROIs examined (Fig. 6a).

## Discussion

The aim of this study was to compare two tau-specific PET tracers in vivo. ^11^C labelling of both tracers allowed their injection into the same patients with AD (prodromal or dementia) on the same day. The two tracers (^11^C-THK5351 and ^11^C-PBB3) differed in both load and regional distribution pattern of binding in the AD brain. Moreover, they displayed different patterns of associations with other markers of the disease, including markers of amyloid-beta deposition, cognitive impairment, CSF tau and medial temporal atrophy.

### Distinctive binding properties of the tau-specific tracers

^11^C-THK5351 showed overall substantially higher binding than ^11^C-PBB3, in agreement with in vitro autoradiography observations [6]. While the regional distribution of ^11^C-THK5351 closely matched the classical distribution pattern of tau pathology [3, 4], the same did not apply for ^11^C-PBB3. ^11^C-PBB3 bound only minimally in the medial temporal lobe, an area known for its abundance of tau pathology early in the AD trajectory. It was somewhat surprising that there was no relationship between the binding of ^11^C-THK5351 and ^11^C-PBB3 either in the temporal lobe or across the whole cortex. While the sample was small and thus only strong relationships between modalities could be detected, the scatterplots indicated a lack of relationship between the two. Altogether, the differences in regional distribution and the lack of correlation between ^11^C-THK5351 and ^11^C-PBB3 binding indicate differences in the molecular targets of the tracers. This may be a result of their different chemical structures and is in agreement with findings of in vitro studies that directly compared the binding characteristics of the three most prominent tau-specific tracers (THK5351, AV-1451 and PBB3) and showed distinct binding sites for the tracers [5, 6]. Nevertheless, the complexity of tau pathology, in terms of the isoforms affected (three and four repeat tau), conformations adopted (paired helical, straight and twisted tau filaments) and types of deposits formed (e.g. neurofibrillary tangles, neuritic plaques, neuropil threads, glial tau deposits), does not preclude the possibility that both ^11^C-THK5351 and ^11^C-PBB3 bind to tau – although to different specific targets, as indicated previously by in vitro results [6].

### Differential relationship with amyloid-beta

The similarities between ^11^C-PBB3 and ^11^C-AZD2184 in terms of regional distribution, especially in the temporal lobe, as well as the very strong regional correlations in tracer binding could potentially raise the question as to the molecular target of ^11^C-PBB3. Furthermore, the facts that ^11^C-PBB3 and ^11^C-AZD2184 have similar chemical structures, although the linker domains differ in length, and have common off-target signals in the dural venous sinuses, add to this uncertainty. Similarities in the molecular targets of ^11^C-PBB3 and ^11^C-AZD2184 indicate limited specificity of ^11^C-PBB3 for tau and a potential binding affinity of the tracer for the more abundant (in the AD brain) amyloid-beta. However, early in vitro evidence to date excludes this possibility [9], although more detailed in vitro competition studies are lacking for further validating the tracer specificity. Additionally, an earlier study that focused on a generally older, more severely affected sample of patients did not show a relationship between ^11^C-PBB3 binding and amyloid-beta burden globally, indicating that the latter relationship could occur only in the early stages of the disease or in distinct brain regions [10]. Indeed, in our study, the regional correlations between ^11^C-PBB3 and ^11^C-AZD2184 binding were limited to the temporal and occipital lobes. The latter areas are well known as the richest areas for neuritic plaques [41] (i.e. dense-core amyloid-beta plaques surrounded by tau-rich dystrophic neurites [42]) in the AD brain. Based on this evidence, it is conceivable that ^11^C-PBB3 binds preferentially to tau deposits located in close proximity to the abundant amyloid-beta plaques in the early symptomatic stages of AD examined in this study, while ^11^C-THK5351 appears to bind to a wider range of tau deposits [43], based on the regional distribution of the tracer. This hypothesis, however, remains to be proven with thorough ante-/post-mortem investigations.

### Differential relationships with cognitive performance and other disease markers

A substantial overlap was observed in terms of ^11^C-THK5351 and ^11^C-PBB3 binding between prodromal and dementia stage AD, although the more detailed neuropsychological evaluation that was employed highlighted that the tracers were able to track the underlying cognitive impairment. More specifically, both tau tracers were similarly correlated with MMSE score, as found in previous studies [10, 13, 44], although this did not apply to a more sensitive measure of global cognition: ^11^C-THK5351 was more sensitive to declines in FSIQ than ^11^C-PBB3. This is consistent with evidence suggesting that ^11^C-THK5351 binding detects tau deposits that are more closely related to atrophy, consistent with post-mortem observations linking tau pathology to neurodegeneration [45, 46]. Furthermore, the rather close relationship between ^11^C-THK5351, but not ^11^C-PBB3, binding and CSF tau levels, similarly to evidence for another tau tracer (^18^F-AV-1451) [47], suggests that the ^11^C-THK5351 molecular target is more closely related to the soluble tau in CSF than to the tau target of ^11^C-PBB3.

### Age-dependent binding

The strong negative relationship between age and ^11^C-PBB3 binding in the temporal lobe was unexpected in our study, especially because of the limited sample size and the narrow age range of the participants. Exploring the age effect was not one of the aims of this study, but was rather an observation while investigating covariates that could potentially have affected the analyses of correlations with cognitive or atrophy measures. Therefore, this finding should be interpreted with caution and its relevance requires further investigation in a larger sample.

### Tracer characteristics and off-target binding

Recent evidence indicates off-target binding of THK5351 and AV-1451 to monoamine oxidase B (MAO-B) [6, 48–50]. In line with the findings of these studies, we observed increased binding of ^11^C-THK5351 in MAO-B-rich areas (striatum, thalamus, cingulate gyri) [51]. Interestingly, however, extensive binding of ^11^C-PBB3 was also observed in the same areas (i.e. striatum and cingulate gyri, but not thalamus). The low tau pathology levels in the striatum and cingulate gyri together with the abundance of MAO-B in the same areas [3, 4, 51] suggest that the three most prominent tau-specific tracers (THK5351, AV-1451 and PBB3) [52], although chemically different, may show some affinity for MAO-B, which could explain their in vivo off-target binding. Further work is required to investigate the contribution of MAO-B binding to the off-target signal of the existing tau tracers, to determine which ROIs are more heavily affected by this off-target signal, and to examine whether structural similarities between tau fibrils and MAO-B are responsible for the observed interaction of the tracers with MAO-B.

The off-target signal of ^11^C-PBB3 in vascular structures (i.e. choroid plexus and dural venous sinuses) could have important implications for quantifying tracer binding. The high binding of ^11^C-PBB3 in the hippocampus that has previously been reported in images from conventional, relatively low-resolution PET systems [9] can now be attributed to spill-over from the intense ^11^C-PBB3 signal in the adjacent choroid plexus. Moreover, the high off-target signal of ^11^C-PBB3 from the dural venous sinuses could complicate the quantification of the tracer because of spill-over of signal from ROIs in close proximity to the sinuses, such as large portions of the parietal, occipital and cerebellar cortices. As an example, the use of the cerebellar cortex (affected by spill-over) as a reference region for ^11^C-PBB3 could prove problematic and lead to underestimation of tracer binding and even negative binding values, especially in ROIs with relatively poor binding. The recognition of off-target binding of the existing tracers and the complications that this binding could cause for quantification of tracer binding is of particular interest as the tracers are employed in ever larger cohorts.

### Limitations

Although the homogeneous sample of patients with AD (prodromal or dementia) in this study was adequate in terms of size with respect to the main aim, the head-to-head comparison of the binding properties of the two tau-specific tracers, it limits the generalizability of the findings when describing the relationships with different biomarkers. Therefore, although we can reach conclusions about differential relationships between the tracers and the different markers of the disease, we cannot exclude the possibility that the relationships that did not reach the threshold for statistical significance were not substantial. More specifically, this study was not designed to refute evidence found in earlier studies, which used larger sample sizes and therefore had greater power to investigate the exact strength of the relationships between the binding of the tracers and the different markers of the disease. In those studies, moderate correlations were found between (a) binding of tracers of the THK family and local amyloid-beta deposition in selected regions, (b) binding of ^11^C-PBB3 and whole-brain grey matter atrophy, and (c) binding of both families of tracers with measures of episodic memory [10, 11, 13, 44]. Lastly, an important limitation of this study lies in the characteristics of the study sample – all participants were relatively young, apolipoprotein E ε4 carriers and had a clear AD-consistent CSF profile – which could limit substantially the generalizability of our findings in the diverse population of patients undergoing cognitive assessment in the clinical setting.

### Conclusion

The load and regional distribution of ^11^C-THK5351 and ^11^C-PBB3 binding suggest different molecular targets for the two tracers, with no similarities observed between them, apart from the common off-target signal from MAO-B-rich areas. The ^11^C-THK5351 pattern fitted best with the expected distribution of tau pathology in AD and related more closely to markers of CSF tau, medial temporal atrophy and cognitive impairment. In contrast, and based on the strong relationships with the amyloid-beta tracer, we suggest that ^11^C-PBB3 could, in the early symptomatic stages of AD, show preferential binding to tau deposits spatially related to amyloid-beta, which could explain its limited association with more downstream markers of the disease (i.e. neurodegeneration and cognitive impairment).

## Electronic supplementary material

ESM 1 (DOCX 3710 kb)
