# Supplementary material for: Dual tracer tau PET imaging reveals different molecular targets for 11C-THK5351 and 11C-PBB3 in the Alzheimer brain
Source: Eur J Nucl Med Mol Imaging. 2018 May 12;45(9):1605–17. doi: 10.1007/s00259-018-4012-5 (PMC6061462; doi:10.1007/s00259-018-4012-5)
Supplement: Supplementary file 1 — (DOCX 3710 kb) [file 259_2018_4012_MOESM1_ESM.docx]

**Online Resources**

Title: Dual tracer tau PET imaging reveals different molecular targets for ^11^C-THK5351 and ^11^C-PBB3 in the Alzheimer brain

**Authors:** Konstantinos Chiotis ^1^; Per Stenkrona ^2^; Ove Almkvist ^1,3,4^; Vladimir Stepanov ^2^; Daniel Ferreira ^5^; Ryosuke Arakawa ^2^; Akihiro Takano ^2^; Eric Westman ^5^; Andrea Varrone ^2^; Nobuyuki Okamura ^6,7^; Hitoshi Shimada ^8^; Makoto Higuchi ^8^; Christer Halldin ^2^; Agneta Nordberg ^1,3^

Affiliations: ^1^Dept of Neurobiology, Care Sciences and Society, Center for Alzheimer Research, Translational Alzheimer Neurobiology, Karolinska Institutet, Stockholm, Sweden; ^2^Department of Clinical Neuroscience, Center for Psychiatric Research, Karolinska Institutet and Stockholm County Council, Stockholm, Sweden; ^3^Theme Aging, Karolinska University Hospital, Stockholm, Sweden; ^4^Department of Psychology, Stockholm University, Stockholm, Sweden; ^5^Dept of Neurobiology, Care Sciences and Society, Center for Alzheimer Research, Division of Clinical Geriatrics, Karolinska Institutet, Stockholm, Sweden; ^6^Cyclotron and Radioisotope Center, Tohoku University, Sendai, Japan; ^7^Division of Pharmacology, Faculty of Medicine, Tohoku Medical and Pharmaceutical University, Sendai, Japan; ^8^National Institute of Radiological Sciences, National Institutes for Quantum and Radiological Science and Technology, Chiba, Japan.

Corresponding Author: Agneta Nordberg, MD, PhD, professor

Karolinska Institutet,

Dept. NVS, Center for Alzheimer Research,

Division of Translational Alzheimer Neurobiology,

Novum 5th floor, 141 57 Huddinge, Sweden

Mail: Agneta.K.Nordberg@ki.se

Phone: +46 8 585 854 67; Fax: +46 8 585 854 70


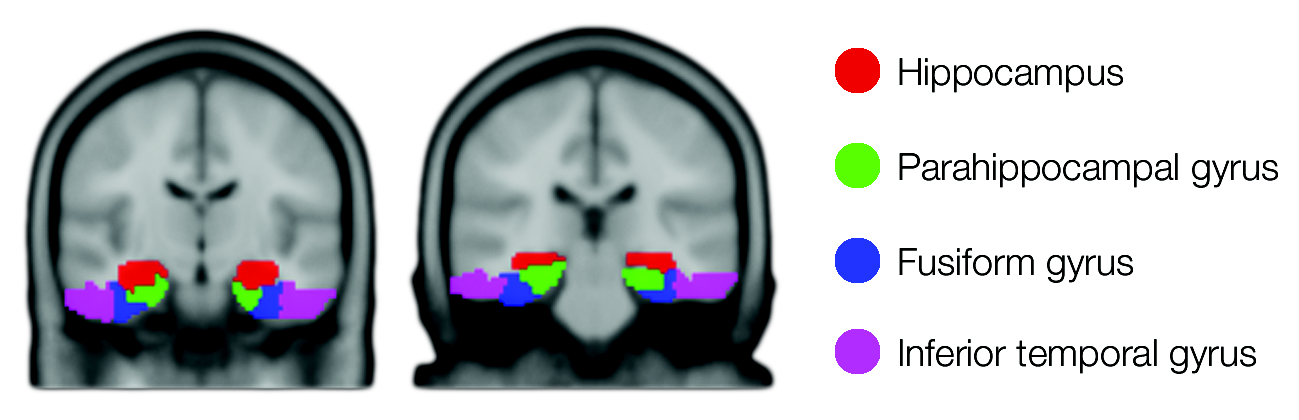


Online Resource 1. Illustration of the sub-regions located in the temporal lobe [[1](#_ENREF_1)], which were used for quantifying the tracer binding. The sub-regions were masked for the individual patient's grey matter in order to show individual grey matter regions of interest, prior to quantifying the tracer binding.

#

Online Resource 2. Scatterplots illustrating the validity of voxel-wise quantification of ^11^C-THK5351 and ^11^C-PBB3 binding (BP_ND_) in nine patients with Alzheimer’s disease using the wavelet-aided parametric imaging (WAPI) method [[2](#_ENREF_2)], versus region-based quantification using the reference Logan graphical method and the original multi-linear reference tissue model (MRTM_O_), as described earlier [[3](#_ENREF_3), [4](#_ENREF_4)]. The results presented in this figure are employing partial volume effect uncorrected data.

Online Resource 3. Boxplots illustrating the regional quantification of the binding of ^11^C-THK5351 (tau), ^11^C-PBB3 (tau) and ^11^C-AZD2184 (amyloid-beta) in patients with Alzheimer’s disease (prodromal or dementia; n=9) after the application of partial volume effect correction (using the geometric transfer matrix method) to the dynamic PET data [[5](#_ENREF_5)]. Open circles stand for patients with prodromal AD and black dots for patients with AD dementia. The horizontal line is representing the median value, the two hinges indicate the first and third quartiles while the whiskers mark the whole range of values excluding potential outliers. Braak I-VI = Regions of interest roughly matching the neuropathological Braak staging system for neurofibrillary tangle pathology, for demonstration purposes only [[6](#_ENREF_6)]; Fus = fusiform gyrus; Hipp = hippocampus; Inf temp = inferior temporal gyrus; Lat. occ = lateral occipital cortex; Med. occ. = medial occipital cortex; Phipp = parahippocampal gyrus.


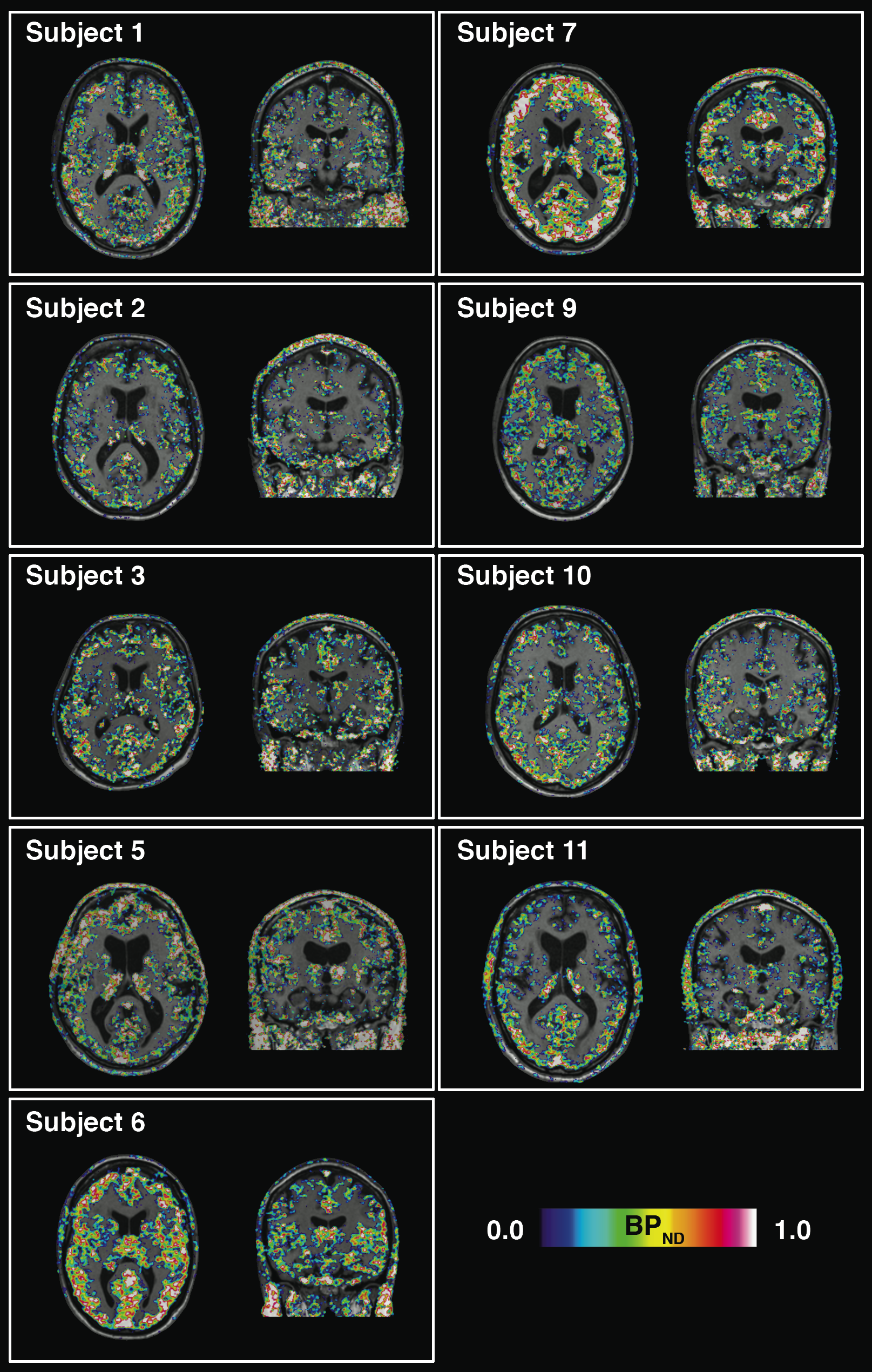


Online Resource 4. High resolution binding potential (BP_ND_) ^11^C-PBB3 PET images of all patients with Alzheimer’s disease (prodromal or dementia; n=9) included in the study illustrating the off-target binding of ^11^C-PBB3 in the choroid plexus in the lateral ventricles, including the choroid plexus of the inferior horn of the lateral ventricles which is adjacent to the hippocampal formation. The voxel-wise modelling of the ^11^C-PBB3 PET images was performed with the use of the wavelet-aided parametric imaging method. The results presented in this figure are employing partial volume effect uncorrected data.

Online Resource 5. Time-activity curves describing the kinetics of ^11^C-THK5351 and ^11^C-PBB3 in the temporal and cerebellar cortices of all patients with Alzheimer’s disease (prodromal or dementia; n=9) included in the study. The results presented in this figure are employing partial volume effect uncorrected data.

# References

1. Tzourio-Mazoyer N, Landeau B, Papathanassiou D, Crivello F, Etard O, Delcroix N, et al. Automated anatomical labeling of activations in SPM using a macroscopic anatomical parcellation of the MNI MRI single-subject brain. NeuroImage. 2002;15:273-89. doi:10.1006/nimg.2001.0978.

2. Cselenyi Z, Olsson H, Farde L, Gulyas B. Wavelet-aided parametric mapping of cerebral dopamine D2 receptors using the high affinity PET radioligand [11C]FLB 457. NeuroImage. 2002;17:47-60.

3. Ichise M, Liow JS, Lu JQ, Takano A, Model K, Toyama H, et al. Linearized reference tissue parametric imaging methods: application to [11C]DASB positron emission tomography studies of the serotonin transporter in human brain. Journal of cerebral blood flow and metabolism : official journal of the International Society of Cerebral Blood Flow and Metabolism. 2003;23:1096-112. doi:10.1097/01.WCB.0000085441.37552.CA.

4. Jonasson M, Wall A, Chiotis K, Saint-Aubert L, Wilking H, Sprycha M, et al. Tracer Kinetic Analysis of (S)-(1)(8)F-THK5117 as a PET Tracer for Assessing Tau Pathology. Journal of nuclear medicine : official publication, Society of Nuclear Medicine. 2016;57:574-81. doi:10.2967/jnumed.115.158519.

5. Rousset OG, Ma Y, Evans AC. Correction for partial volume effects in PET: principle and validation. Journal of nuclear medicine : official publication, Society of Nuclear Medicine. 1998;39:904-11.

6. Alafuzoff I, Arzberger T, Al-Sarraj S, Bodi I, Bogdanovic N, Braak H, et al. Staging of neurofibrillary pathology in Alzheimer's disease: a study of the BrainNet Europe Consortium. Brain pathology. 2008;18:484-96. doi:10.1111/j.1750-3639.2008.00147.x.
